# “I Can’t Lift My Legs”: An Interesting Presentation Leading to the Diagnosis of Anti-synthetase Syndrome

**DOI:** 10.7759/cureus.87570

**Published:** 2025-07-08

**Authors:** Javed A Hussain, Chinju Antony, Madan Thirunavukarasu, Athar Yasin

**Affiliations:** 1 Emergency Medicine, Peterborough City Hospital, Peterborough, GBR

**Keywords:** anti-jo 1 antibodies, antisynthetase syndrome (ass), creatinine kinase, cyclophosphamide treatment, ild interstitial lung disease

## Abstract

Anti-synthetase syndrome (ASS) is an autoimmune disorder that often manifests through lung involvement such as interstitial lung disease, inflammation of the muscles, and symptoms affecting the joints, including pain or arthritis. Identifying ASS involves a multidisciplinary effort between rheumatologists and lung specialists, using a combination of blood work, imaging techniques, and, in some cases, biopsies of muscle or lung tissue to reach a diagnosis. Treatment usually involves corticosteroids along with other immunosuppressive medications. In this case, we explore the diagnostic journey of a 37-year-old patient who initially presented with an unusual complaint: difficulty lifting his leg, alongside a range of other symptoms. This case highlights the complexity and challenges involved in identifying anti-synthetase syndrome.

## Introduction

The idiopathic inflammatory myopathies (IIM) consist of rare, heterogeneous autoimmune disorders that present with marked proximal and symmetric muscle weakness, except for distal and asymmetric weakness in inclusion body myositis (IBM) [1]. IIM can be classified into several subgroups: dermatomyositis (including amyopathic dermatomyositis), anti-synthetase syndrome, immune-mediated necrotizing myopathy, inclusion body myositis, polymyositis, and overlap myositis [2]. All of these inflammatory myopathies, excluding IBM, present with subacute symmetrical proximal weakness. Patients may complain of increasing difficulty rising from a chair, climbing stairs, washing their hair, or hanging washing [3]. 

Anti-synthetase syndrome (ASS) is an autoimmune condition, characterized by antibodies directed against an aminoacycl transfer RNA synthetase [4]. The main clinical features of ASS are fever, myositis, polyarthritis, interstitial lung disease, 'mechanic’s hands' (thick, cracked skin usually on the palms and radial surfaces of the digits), Raynaud phenomenon, Gottron’s papules (lesions on metacarpophalangeal and interphalangeal joint areas) [5]. Anti-synthetase antibodies (ASAb) include: anti-histidyl (anti-Jo-1, being the best known), anti-threonyl (anti-PL-7), anti-alanyl (anti-PL-12), anti-isoleucyl (anti-OJ), anti-glycyl (anti-EJ), anti-asparaginyl (anti-KS), anti-Wa, anti-tyrosil (anti-YRS), anti-phenylalanyl-transfer RNA synthetase (anti-Zo), and anti-signal recognition particles (anti-SRP) [1].

In this case report, we discuss a 37-year-old patient who presented to our emergency department with complaints of muscle weakness and shortness of breath and was found to have an elevated creatine kinase level. We explore the challenges involved in diagnosing anti-synthetase syndrome and discuss the various investigative approaches, including muscle biopsy, MRI, and antibody testing.

## Case presentation

A 37-year-old male patient presented to the emergency department with weakness, progressive shortness of breath, epigastric discomfort, and worsening fatigue over a two-month period. He stated that he had trouble lifting his legs, particularly when trying to climb stairs or get into bed. He reported that his dyspnea, initially exertional, had advanced to the point of occurring even at rest. The fatigue had become profound, significantly impairing his ability to perform daily activities. He had no known past medical history, was a non-smoker, and reported only occasional alcohol consumption.

On examination, he was tachycardic at 110 bpm with reduced oxygen saturation of 93% on room air. He was noted to have 'mechanic's hands'. Auscultation of the lungs revealed bilateral crackles and abdominal examination was unremarkable. Notably, the patient exhibited proximal muscle weakness with a power 3/5 and had difficulty climbing onto the examination bed. Arterial blood gas analysis revealed type 1 respiratory failure with a lactate level of 3.67 mmol/L, and electrocardiography demonstrated sinus tachycardia. Urinalysis was positive for both hematuria and proteinuria.

**Figure 1:**
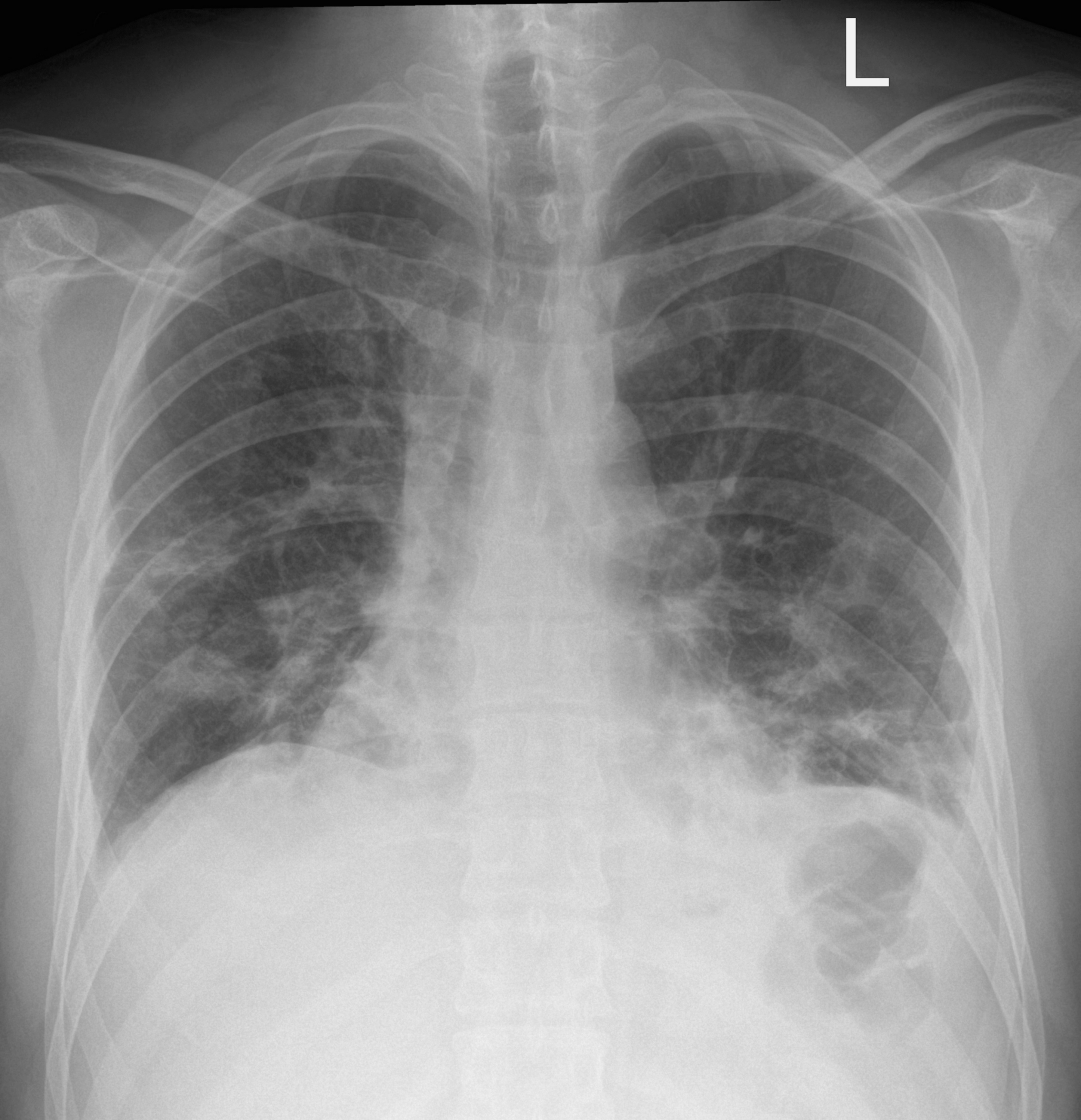
Chest X-ray showing bilateral infiltrative lung changes

A chest X-ray (Figure 1) demonstrated bilateral infiltrative changes along with mediastinal widening. Laboratory investigations (Table 1) revealed elevated levels of C-reactive protein (CRP), creatine kinase (CK), ferritin, and alanine aminotransferase (ALT). The patient was commenced on supplemental oxygen via nasal cannula and received intravenous antibiotics.

**Table 1:** Laboratory results on admission

Parameter	Reference range and units	First day of admission
WBC count	4-11 × 10^9^/L	14.3
Haemoglobin	115-165 g/L	150
Platelet count	150-400 × 10^9^/L	603
Lymphocytes	1.4 – 4.8 x 10^9^/L	2.1
Neutrophils	1.8 – 7.7 x 10^9^/L	11.2
Monocytes	0.1 – 0.8 x 10^9^/L	0.7
Eosinophils	0.1 – 0.6 x 10^9^/L	0.2
Basophils	0 – 0.1 x 10^9^/L	0
Sodium	133-146 mmol/L	130
Potassium	3.5-5.3 mmol/L	5.2
Chloride	95-108 mmol/L	98
Creatinine	45-84 umol/L	87
CRP	<5 mg/L	41
Lactic acid	0.5-2 mmol/L	3.67
ALT	<41 IU/L	554
AST	10-50 IU/L	1031
ALP	30-130 IU/L	119
Total Bilirubin	0-21umol/L	7
Creatinine Kinase	40-320 IU/L	21871
ESR	0-13 mm/h	26
Ferritin	30-400 ug/L	6545
Troponin T	<12 ng/L	1233

Given the nonspecific clinical presentation, radiographic findings, and laboratory abnormalities, a referral to a specialist was made for further diagnostic evaluation and management. The patient provided additional history, stating that for the past six months, he had noticed changes in his health. He mentioned experiencing intermittent abdominal discomfort, occasionally associated with diarrhoea, and a reduced appetite. The shortness of breath began two months ago but has worsened to the point where he had to quit his job. Over the past two weeks, he has noticed significant weakness in his legs, to the extent that he needs to use his arms to lift his legs when getting into bed. He also shared that his mother had pancreatic cancer, but there is no other significant family history of medical conditions.

**Figure 2:**
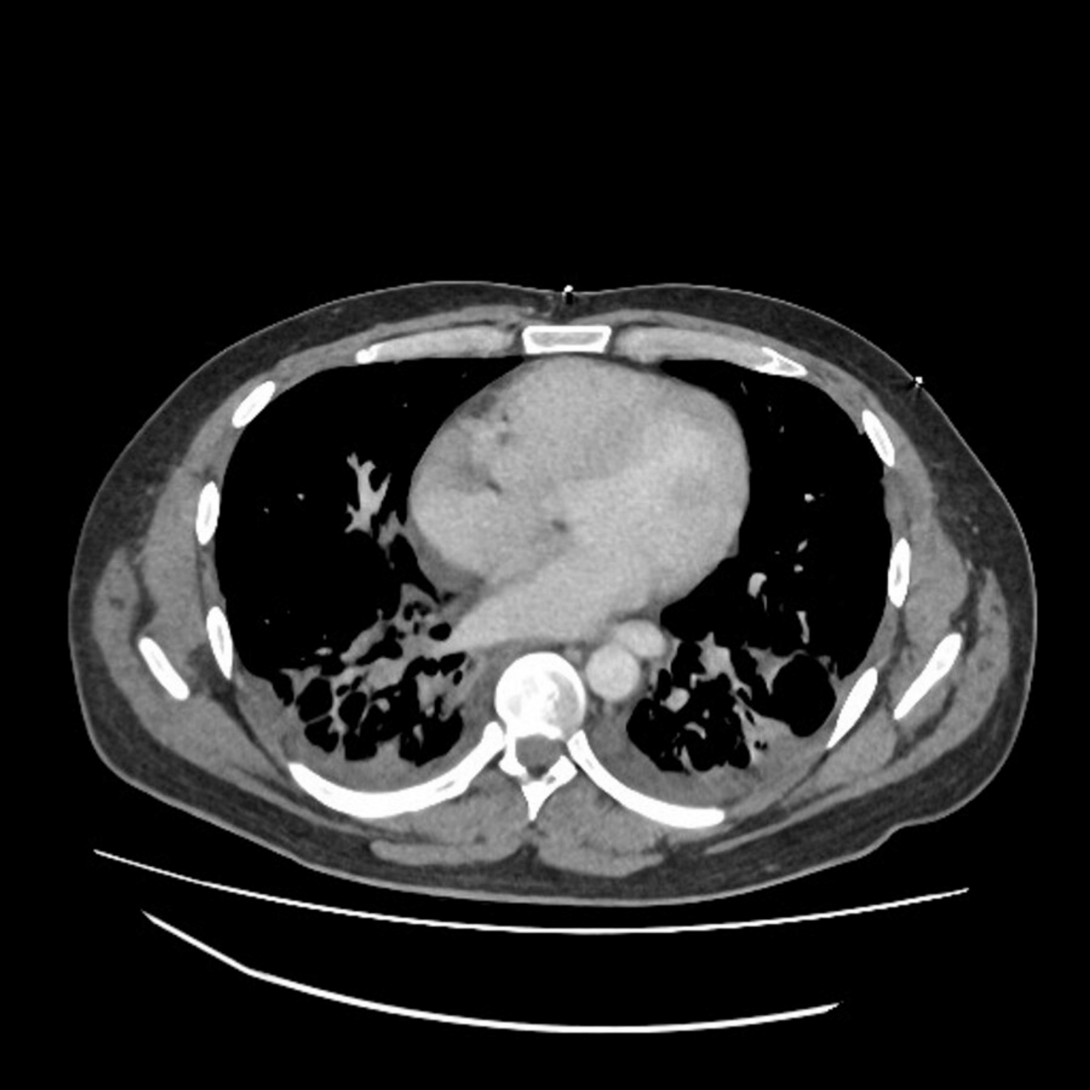
CT Chest showing bilateral symmetrical inflammatory changes in the lungs with small bilateral pleural effusions.

Rheumatology was involved in the care, and a provisional diagnosis of autoimmune inflammatory myositis was made. The patient was started on intravenous steroids and was transferred to the intensive care unit due to worsening oxygen saturations. A whole-body CT scan (Figure 2) revealed bilateral symmetrical inflammatory changes in the lungs with bilateral pleural effusions, but everything else appeared unremarkable. EMG showed myopathy with fibrillations on the proximal leg muscles. 

**Figure 3:**
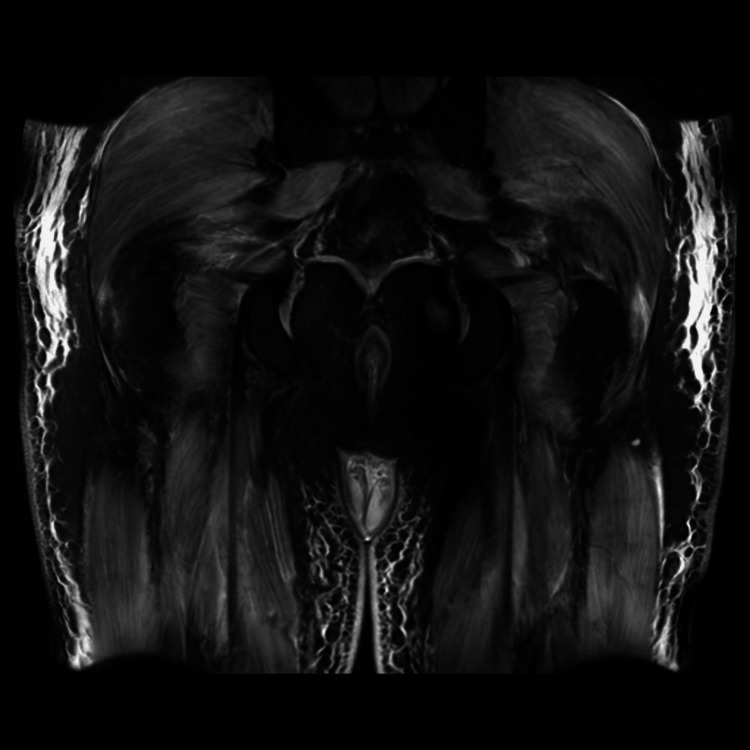
MRI pelvis showing extensive diffuse muscle oedema in both thighs and gluteal muscles.

**Figure 4:**
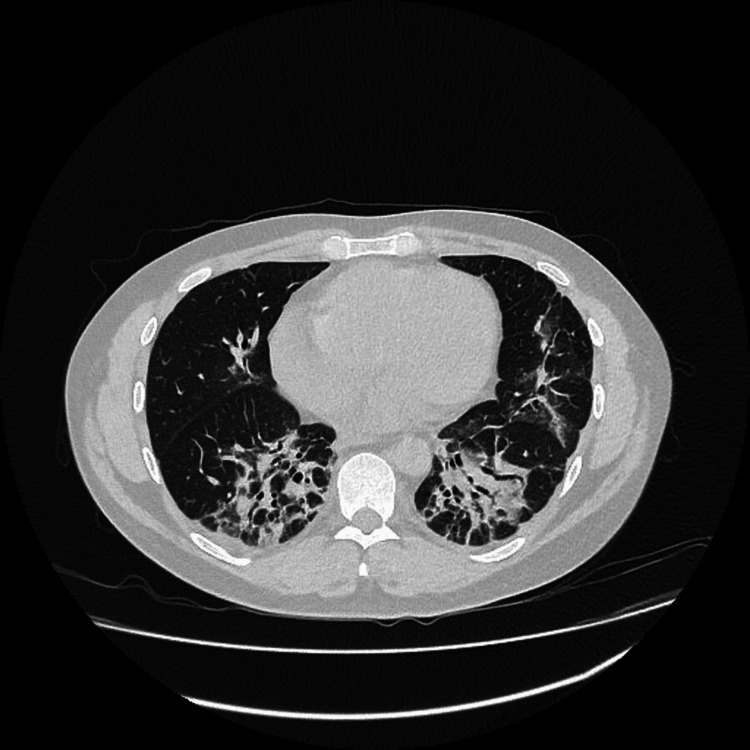
A high-resolution CT scan shows organizing pneumonia pattern and appears to be resolving

The patient's troponin levels were elevated. However, given the absence of chest pain and ECG changes, the cardiology team believed this was more likely a type 2 event rather than acute coronary syndrome and was probably due to myocarditis. An echocardiogram was performed and showed normal results. An MRI of the four limbs (Figure 3) revealed extensive diffuse muscle edema, consistent with bilateral extensive myositis. An MRI of the spine was conducted to rule out other factors contributing to the weakness, which came back normal. Gluteal biopsy was done and showed perifascicular necrosis, sarcolemmal MHC-1 expression, and inflammation in the perimysium which is suggestive of dermatomyositis. Antibody screen showed positive for anti-Ro and anti-Jo 1 (Table 2). Based on the clinical presentation and confirmation through antibody testing, the patient was diagnosed with ASS. A high-resolution CT scan (Figure 4) revealed a pattern consistent with organizing pneumonia, which is a form of interstitial lung disease.

**Table 2:** Blood tests done as part of workup for ASS ASS: anti-synthetase syndrome; ANCA: antineutrophil cytoplasmic antibody; anti-CPP: anti-cyclic citrullinated peptide

Parameter	Result	Reference Range
Rheumatoid factor	<12 IU/mL	<14
Beta 2 microglobulin	13.1 mg/L	0.8 - 2.2
Anti-nuclear antibody	7.7	<0.7
Neutrophil cytoplasmic antibody	Negative	
Protein electrophoresis	Normal	
Anti-CCP antibodies	Negative	
Glomerular basement membrane antibody	Negative	
Smooth muscle antibodies	Negative	
Mitochondrial antibodies	Negative	
Myeloperoxidase ANCA	<0.2 IU/mL	<3.5
Proteinase 3 (PR3) ANCA	<0.6 IU/mL	<2.0
Extractable nuclear antibody	9.2 U/mL	<0.7
dsDNA antibody	Negative	
Ro (SSA) antibody	144.0 U/mL	<7
La (SSB) antibody	<0.4 U/mL	<7
U1 RNP antibody	1.2 U/mL	<5
Sm (Smith) immunoglobulin G antibody	<0.7 U/mL	<7
Scl-70 (topoisomerase-1)antibody	<0.6 U/mL	<7
Jo-1 (aminoacyl-tRNA histidyl)	62.0 U/mL	<7
Centromere antibody	<0.4 U/mL	<7

The patient was started on the Euro-Lupus regimen of cyclophosphamide. The patient improved symptomatically and was discharged with biweekly cyclophosphamide infusions and is currently scheduled to follow-up with rheumatology.

## Discussion

According to studies, ASS is predominantly affects the female population; the mean female/male ratio of AS syndrome is 2:1 (in some studies, this ratio is as great as 13:1) [6]. However, this does not preclude its occurrence in males, as demonstrated by our patient.

**Figure 5:**
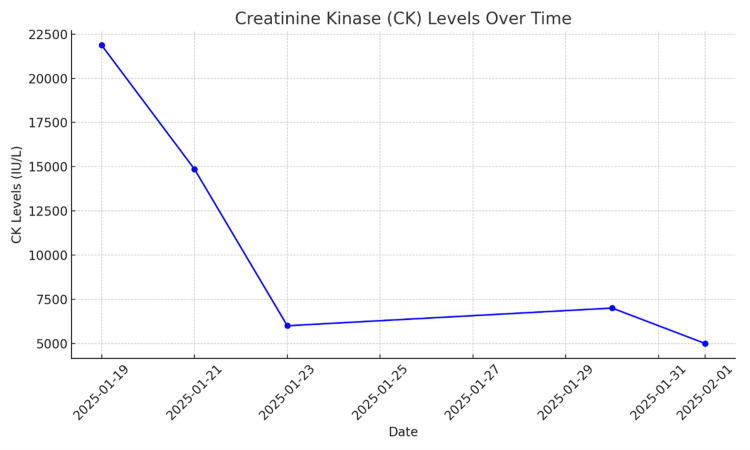
The graph above illustrates the trend of creatine kinase levels throughout the patient's hospital stay.

Early diagnosis is difficult because the clinical presentation is varied and often nonspecific. Clinically milder disease may escape detection, and many general practitioners lack familiarity with this syndrome and consequently do not recognize it [7]. Our patient presented with an unusual and longstanding clinical picture. Initially, it was challenging to establish a clear link between his proximal muscle weakness and the recent onset of dyspnoea. As clinicians in the emergency department, it was the elevated creatine kinase levels (Figure 5), the presence of mechanic's hands, and proximal limb weakness that raised our suspicion of an underlying inflammatory myopathy, prompting us to seek input from the medical and rheumatology teams.

Gusdorf et al. (2018) reported that mechanic's hands were present in approximately 40% of patients with ASS [8]. This suggests a strong association between the presence of mechanic’s hands and a diagnosis of ASS, as observed in our patient.

Interstitial lung disease (ILD) is especially frequent in ASS, reported in 70-90% of cases, and drives the prognosis [9]. Initial chest X-ray and CT scan showed inflammatory changes in the lungs. A follow-up high-resolution CT was planned after four weeks to assess for progression, which revealed features consistent with an organizing pneumonia pattern, which is associated with interstitial lung disease. ASS patients with associated anti-Ro/SS-A antibodies seem to be predisposed to the development of a more severe ILD [10]. Our patient tested positive for anti-Ro antibodies, which indicates a higher risk of developing severe ILD.

The treatment, however, includes anti-inflammatory and immunosuppressive drugs. Corticosteroids are the first-line agents with immunosuppressants such as azathioprine, mycophenolate mofetil, tacrolimus, rituximab, and cyclophosphamide as an add-on therapy for pulmonary and muscle involvement [4]. Our patient was commenced on prednisolone as soon as ASS was suspected. Following confirmation of the diagnosis through positive anti-Jo-1 antibodies and a gluteal muscle biopsy, cyclophosphamide infusions were initiated. Although corticosteroids are commonly employed as initial monotherapy in ASS, pulmonary disease frequently recurs during dose tapering, necessitating the use of adjunctive immunosuppressive therapies. Frequently used adjunctive agents include azathioprine, mycophenolate mofetil, tacrolimus, rituximab, and cyclophosphamide, though there is little consensus about which agent is preferred [10]. We initiated treatment with the Euro-Lupus intravenous cyclophosphamide regimen, which involves administering 500 mg every two weeks for a total of six doses.

Although muscle biopsy is infrequently necessary to establish a diagnosis of ASS, it may be essential for differentiating ASS from other etiologies of myopathy, including muscular dystrophies, metabolic disorders, infections, and toxin- or drug-induced myopathies [11]. A gluteal biopsy was done for our patient, which showed prominent perifasicular lymphocytic inflammation, fibre regeneration, atrophy, and necrosis with internalisation of nuclei, which are suggestive of dermatomyositis.

## Conclusions

ASS remains a relatively underrecognized condition, which can contribute to delays in diagnosis. Given its rarity and variable clinical presentation, ASS often requires a high index of suspicion to prompt further diagnostic evaluation. The diagnosis typically involves a comprehensive workup, including MRI, muscle biopsy, and serological testing for specific autoantibodies, such as anti-Jo-1 and other anti-aminoacyl-tRNA synthetase antibodies. Early recognition of ASS is critical, as the disease can progress rapidly, potentially leading to significant morbidity. Patients diagnosed with ASS require close clinical monitoring, not only due to the risk of disease exacerbation but also because they are at increased risk for developing associated conditions, such as interstitial lung disease and malignancy. Vigilant surveillance and a multidisciplinary approach are essential to optimize patient outcomes and manage the complex clinical manifestations of this syndrome.
